# Effect of Sintering Temperature on Densification, Microstructure, and Corrosion Behavior of Ti6Al4V/20Cu Composites Fabricated by Powder Metallurgy

**DOI:** 10.3390/ma19142979

**Published:** 2026-07-10

**Authors:** Victor Manuel Solorio, Hector Javier Vergara-Hernández, Elena Mihalcea, Julio Villalobos-Brito, Francisco Alvarado-Hernandez, Jose Luis Cabezas-Villa, Gilberto González-Gómez, Mario Misael Machado-López, Luis Olmos

**Affiliations:** 1 División de Estudios de Posgrado e Investigación, Tecnológico Nacional de Mexico/I.T. Morelia, Av. Tecnológico #1500, Colonia Lomas de Santiaguito, Morelia C.P. 58120, Mexico; d12121802@morelia.tecnm.mx (V.M.S.); hector.vh@morelia.tecnm.mx (H.J.V.-H.); julio.vb@morelia.tecnm.mx (J.V.-B.); mario.ml@morelia.tecnm.mx (M.M.M.-L.); 2 Unidad Académica de Ingeniería I, Universidad Autónoma de Zacatecas, Zacatecas C.P. 98000, Mexico; emihalcea@itmorelia.edu.mx (E.M.); ingenierofah@uaz.edu.mx (F.A.-H.); 3 Instituto de Investigaciones en Ciencias de la Tierra, Universidad Michoacana de San Nicolás de Hidalgo, Fco. J. Mujica S/N, Morelia C.P. 58060, Mexico; luis.cabezas@umich.mx; 4 Departamento de Ingeniería Química Tecnológico Nacional de México/I.T.Celaya, Tecnológico Nacional de Mexico en Celaya, Celaya C.P. 38010, Mexico; gilberto.gonzalez@itcelaya.edu.mx

**Keywords:** liquid-phase sintering, Ti6Al4V, copper alloying, densification, microstructure, corrosion resistance, biomedical implants

## Abstract

**Highlights:**

- Effect of temperature on densification: Relative density increased progressively from 78.6% at 900 °C to 98.1% at 1100 °C through liquid-phase sintering.
- Microstructural evolution: Increasing the temperature facilitated the transition from fragmented Ti-Cu dendritic structures to refined globular intermetallics (TiCu and Ti_2_Cu).
- Copper diffusion and phase evolution: Increasing temperature promoted enhanced copper diffusion into the Ti matrix together with the formation of TiCu and Ti_2_Cu intermetallic phases, resulting in a more homogeneous microstructure.
- Mechanical property enhancement: The microhardness of the composite increased systematically by 50%, from 313 HV to 473 HV, due to porosity reduction and intermetallic phase hardening.
- Electrochemical performance: Consolidation at high temperatures optimized the microstructure, a key factor in assessing corrosion resistance in biomedical implant applications.

**Abstract:**

Copper alloying of Ti6Al4V via liquid-phase sintering (LPS) is a promising route to enhance densification and mechanical properties for biomedical implants. This study investigates the effect of sintering temperature (900–1100 °C) on the densification, microstructure, and electrochemical behavior of Ti6Al4V–20 wt.% Cu composites. Samples were fabricated via pressureless sintering, maintaining a constant relative green density of 72.7%. The results show that the relative density increased progressively from 78.6% at 900 °C to 98.1% at 1100 °C. Microstructural analysis revealed a transition from fragmented Ti-Cu dendritic structures to refined globular intermetallic, with enhanced copper diffusion into the α-Ti matrix above 1000 °C, accompanied by the formation of TiCu and Ti_2_Cu intermetallic phases. Correspondingly, microhardness increased systematically from 313 HV to 473 HV, correlated with reduced porosity and intermetallic reinforcement. Electrochemical tests in Ringer’s solution indicated that while higher temperatures improve structural integrity, the distribution of Cu-rich phases significantly influences corrosion kinetics. These findings demonstrate that sintering at 1100 °C optimizes the densification–microstructure relationship, providing a technical basis for the development of high-performance Ti-based composites. Based on previous studies of Ti–Cu systems, these materials may exhibit antibacterial activity, although no biological or antibacterial tests were performed in the present work.

## 1. Introduction

Titanium alloys, particularly Ti6Al4V, are extensively utilized in aerospace, biomedical, and high-performance engineering applications due to their exceptional strength-to-weight ratio, corrosion resistance, and biocompatibility. However, their limited wear resistance and absence of inherent antibacterial properties constrain their broader application in biomedical implants and components subjected to tribological stress. To address these limitations, alloying Ti6Al4V with copper (Cu) has emerged as a promising strategy to potentially impart antibacterial functionality while simultaneously enhancing mechanical properties through the formation of Ti–Cu intermetallic phases such as Ti_2_Cu, TiCu, Ti_3_Cu_4_, and metastable TiCu_3_ [1,2,3,4,5]. Liquid-phase sintering (LPS) is a cost-effective powder metallurgy route for fabricating Ti6Al4V–Cu composites with controlled microstructures and near-net-shape geometries. During LPS, the transient liquid phase generated by Cu melting (above 1084 °C) promotes rapid densification through capillary-driven liquid redistribution, particle rearrangement, and enhanced solid–liquid diffusion. According to the Ti–Cu binary phase diagram, the eutectic reaction occurs at approximately 875 °C, with a eutectic composition near 7 wt% Cu, thereby facilitating the formation of multiple intermetallic phases during cooling. Recent investigations have demonstrated that LPS of Ti–Cu systems achieve relative densities exceeding 96% at temperatures 80–100 °C lower than those of conventional solid-state sintering, attributed to the high diffusivity of Cu in the Ti matrix and the formation of low-viscosity Cu-rich liquid phases [1,6,7,8,9]. The sintering temperature critically governs phase transformation pathways, microstructural evolution, and densification kinetics in Ti6Al4V–Cu composites. German’s mechanistic model of titanium sintering identifies three distinct densification regimes: (i) initial neck formation and α-β interface-enhanced diffusion at 800–1000 °C, (ii) rapid densification during the α→β transformation near 950 °C, and (iii) final-stage densification via β-phase grain boundary and volume diffusion above 1000 °C. In Ti–Cu systems, the addition of Cu modifies these mechanisms by introducing eutectoid transformations (β → α + Ti_2_Cu at 792 °C) and eutectic solidification reactions that generate complex microstructures comprising globular Ti–Cu intermetallics embedded in an α-Ti matrix [10,11,12]. Microstructural characterization studies reveal that increasing sintering temperature from 900 to 1100 °C promotes the fragmentation of dendritic Ti–Cu structures into refined globular morphologies through a coalescence mechanism driven by capillary forces and plastic deformation at grain boundaries. Konieczny et al. demonstrated that prolonged heat treatment at 900 °C transforms multi-phase Ti–Cu intermetallic layers (Ti_2_Cu, TiCu, Ti_3_Cu_4_, Ti_2_Cu_3_, TiCu_4_) into predominantly TiCu compounds due to thermodynamic favorability (ΔGf = −10.7 kJ/mol for TiCu). Furthermore, two-step pressureless sintering strategies have achieved simultaneous densification and grain refinement in Ti6Al4V alloys, yielding densities of 99% with β-grain sizes below 100 μm [7,13,14,15]. Electrochemical assessments indicate that Ti–Cu alloys exhibit superior corrosion resistance compared to pure Ti6Al4V, with corrosion rates below 0.005 mm/year and polarization resistances exceeding 1 × 10^5^ Ω·cm^2^ in simulated body fluid. Previous studies have reported that Ti–Cu alloys can exhibit enhanced passivation and antibacterial activity due to the combined effects of passive oxide films and controlled copper-ion release. However, antibacterial performance was not evaluated in the present study. Recent reviews emphasize the dual functionality of Ti–Cu alloys, which balance mechanical performance with biological activity, positioning them as promising candidates for next-generation orthopedic and dental implants [5,15,16,17,18,19,20,21]. Despite extensive research on Ti–Cu systems, systematic correlations between sintering temperature, phase evolution, densification mechanisms, and combined mechanical–electrochemical performance remain incompletely understood. This work investigates the effect of sintering temperature (900–1100 °C) on the densification behavior, microstructural transformations, and corrosion resistance of Ti6Al4V–20 wt.% Cu composites fabricated via liquid phase sintering. Through integrated characterization using X-ray diffraction (XRD), scanning electron microscopy (SEM) with energy-dispersive X-ray spectroscopy (EDS), Vickers microhardness testing, potentiodynamic polarization, and electrochemical impedance spectroscopy (EIS), the study elucidates the roles of Cu diffusion, intermetallic phase formation, and microstructural refinement in governing densification kinetics and corrosion performance. The findings provide fundamental insights into processing–structure–property relationships in Ti–Cu alloys, offering guidance for designing high-performance materials for biomedical and structural applications.

## 2. Materials and Methods

### 2.1. Preparation of Powder Mixtures

The starting materials consisted of pre-alloyed spherical Ti6Al4V powders (particle size ≤ 20 µm) and irregularly shaped copper (Cu) powders (particle size ≤ 5 µm, supplied by Sigma-Aldrich, St. Louis, MO, USA). The composites were prepared by mixing Ti6Al4V with 20% by weight of Cu reinforcement. Volume fractions were determined using the rule of mixtures, based on the theoretical densities of Ti6Al4V (*_δ_*Ti64 = 4.45 g/cm) and copper (*_δ_*Cu = 8.96 g/cm^3^). To ensure the structural integrity of the green bodies, 1 wt.% of polyvinyl alcohol (PVA) was added as a binder. The green compacts were prepared by pouring the mixtures into an 8 mm diameter stainless steel die and pressing them at 500 MPa using an Instron 1150 series universal testing machine to achieve a height of 4 mm.

### 2.2. Consolidation and Sintering

The green compacts were prepared by pouring the Ti6Al4V/20Cu powder mixtures into an 8 mm diameter stainless steel die and pressing them at a compaction pressure of 500 MPa using an Instron 1150 series universal testing machine. This process resulted in green compacts with a standardized height of 4 mm. The sintering characterization was conducted to evaluate the effect of temperature on the sintering kinetics, physical properties, and microstructural evolution. The compacts were analyzed using a vertical dilatometer (LINSEIS L75V series, Selb, Germany). The thermal cycles were performed between 900 °C and 1100 °C, with 50 °C increments and a constant heating rate of 10 °C/min. These tests were carried out under a high-purity argon atmosphere with a short dwell time of 5 min at the peak temperature to monitor the onset of densification.

### 2.3. Physical and Mechanical Characterization

#### 2.3.1. Density Measurement

The density of the compacts was determined using the geometric method, which involves precisely measuring the mass and dimensions of each sample before and after sintering. The bulk density (ρb) was calculated from these measurements. The theoretical densities of Ti6Al4V and Cu were taken as 4.45 and 8.96 g cm^−3^, respectively. Using the rule of mixtures, the theoretical density of the Ti6Al4V–20 wt.% Cu composite was calculated as 5.04 g cm^−3^. Relative density was subsequently determined by dividing the measured bulk density by the theoretical composite density. Three specimens were measured for each sintering condition to determine the relative density using the geometric method.

#### 2.3.2. Microhardness Testing

Vickers microhardness (HV) was measured on the polished surfaces of the specimens using a Mitutoyo MVK-HVL (Kawasaki, Japan) microhardness tester. The tests were performed with a load of 200 g and an indentation time of 15 s. To ensure statistical representativeness and to evaluate hardness across the entire surface, indentations were performed at random locations, maintaining sufficient spacing to avoid the influence of prior plastic deformation. A minimum of five indentations per sample were averaged to determine the final microhardness value.

#### 2.3.3. Compression Testing

Mechanical properties, including Young’s modulus and yield strength, were evaluated through compression tests using an Instron 1195 series universal testing machine at a constant displacement rate of 0.5 mm/min. Three samples were tested for each sintering temperature. The stress was calculated by dividing the applied force by the instantaneous area, accounting for the radial growth (barreling) of the specimens during loading. The instantaneous radius and diameter were estimated based on the initial dimensions and the recorded displacement. The resulting force-displacement data were processed to generate stress–strain curves. The Young’s modulus was estimated from the initial linear portion of the compression stress–strain curves using linear regression analysis. Strain values were calculated from the crosshead displacement recorded by the testing machine. Therefore, the reported values should be considered apparent compressive moduli, since no external extensometer or machine-compliance correction was employed. Yield strength was determined from the transition between the elastic and plastic deformation regions. Although this methodology allows comparative evaluation between processing conditions, the absolute modulus values may differ from those reported for fully dense Ti6Al4V alloys measured using standardized tensile testing procedures.

### 2.4. Microstructural and Phase Analysis

#### 2.4.1. Metallographic Preparation

To evaluate the microstructural evolution, the sintered compacts were first examined externally and then internally. Radial cross-sections were obtained using an Accutom cutter equipped with a diamond disc, operating at 1500 rpm to prevent overheating and mechanical deformation that could alter the microstructure. The specimens underwent a rigorous metallographic preparation process: grinding with SiC abrasives of increasing grit sizes (240–2000), followed by polishing to a mirror finish using Leco cloths, utilizing 1 µm alumina as the abrasive medium. Finally, the samples were cleaned in an ultrasonic bath with acetone for 30 min (three 10 min cycles).

#### 2.4.2. Scanning Electron Microscopy (SEM)

Microstructural characterization, morphology, and elemental distribution were analyzed using a Field Emission Scanning Electron Microscope (FE-SEM, Tescan Mira 3, Brno, Czech Republic). Energy-Dispersive X-ray Spectroscopy (EDS) was used to perform chemical mapping and point analyses, focusing on copper distribution and the formation of intermetallic phases within the Ti6Al4V matrix.

#### 2.4.3. X-Ray Diffraction (XRD)

Phase identification and crystalline structure analysis were performed on the polished cross-sections using a Panalytical Empyrean diffractometer with Cu-K\α radiation (λ = 0.154 nm). This analysis was crucial for identifying the phase transformations occurring at sintering temperatures and the specific intermetallic compounds formed in the Ti6Al4V/20Cu system. The diffractometer operated at 30 kV and 30 mA. Measurements were recorded over a 2θ scanning range from 20° to 80°, with a step size of 0.02° and a dwell time of 1.5 s per step.

### 2.5. Electrochemical Evaluation

The corrosion resistance of the Ti6Al4V–20Cu composites was evaluated using a standard three-electrode cell configuration. A Standard Calomel Electrode (SCE) was used as the reference electrode, a graphite rod as the counter electrode, and the sintered samples as the working electrodes. The tests were conducted in simulated physiological media: Hank’s balanced salt solution (synthetic saliva). All measurements were performed at a controlled temperature of 37 ± 0.5 °C. Prior to the electrochemical measurements, the specimens were immersed in the electrolyte for 1200 s to reach a stable Open Circuit Potential (OCP). Linear Sweep Voltammetry (LSV) was performed within a potential range of ±2.0 V at a scan rate of 10 mV/s. Potentiodynamic Polarization (PDP) curves were recorded at a scan rate of 0.01 V/s to determine the corrosion current density (icorr) and corrosion potential (Ecorr). The anodic Tafel slopes were analyzed to evaluate the corrosion activity as a function of the copper reinforcement content and sintering temperature. Electrochemical Impedance Spectroscopy (EIS) was carried out at the OCP using an alternating current (AC) signal of 10 mV, covering a frequency range from 10 kHz to 100 mHz. Nyquist and Bode plots were generated from the EIS data to characterize the stability and protective properties of the passive films formed on the sample surfaces. One electrochemical measurement (potentiodynamic polarization and EIS) was performed for each sintering condition under identical experimental conditions.

## 3. Results and Discussion

### 3.1. Sintering

**Figure 1:**
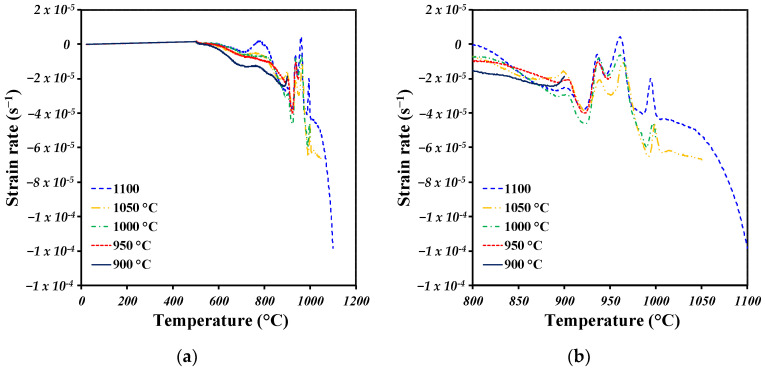
Axial strain during the complete sintering cycle (**a**) and strain rate versus temperature during heating to the sintering plateau (**b**).

The evolution of axial strain during the sintering cycle for Ti64 powder and its Cu-containing composites is shown in Figure 1. All samples exhibit identical thermal behavior initially, characterized by thermal expansion followed by progressive contraction (decreasing strain) up to 6000 s, corresponding to approximately 910 °C. At this critical temperature, the Cu-bearing composites display a subtle inflection point (denoted by the red rectangle in Figure 1a), which becomes more pronounced when the strain rate is plotted against temperature (Figure 1b) [1]. This attenuation of the contraction rate is attributed to the peritectic reaction between Ti and Cu occurring near 925 °C, as predicted by the Ti–Cu binary phase diagram [1,22].

Following this transition, axial strain continues to decrease, but the rate of contraction diverges markedly as a function of Cu content in samples containing 15 and 20 wt. % Cu exhibit substantially accelerated contraction kinetics, whereas composites with 5 and 10 wt.% Cu demonstrate significantly lower contraction rates, only marginally higher than those of pure Ti64. This compositional dependence reveals a fundamental mechanistic transition: at lower Cu concentrations (≤10 wt.%), densification is predominantly governed by solid-state particle rearrangement and neck formation of Ti64 particles, consistent with German’s mechanistic model of titanium sintering [2]. Conversely, at higher Cu loadings (≥15 wt.%), the transient liquid phase generated by the eutectic reaction between Ti and Cu becomes the controlling densification mechanism [4], driving accelerated shrinkage through capillary-induced particle redistribution and enhanced solid–liquid diffusion. This transition is critical to understanding the superior densification efficiency achieved at 1100 °C in the Ti64/20Cu composite, in which liquid-phase sintering mechanisms dominate and relative densities exceed 97% [3,4]. The initial thermal expansion phase reflects conventional lattice expansion in the α-Ti phase; the competing effects of solid-state sintering and phase-transformation-induced volume changes govern the subsequent contraction. The subtle strain-rate inflection near 910 °C likely corresponds to the incipient α→β transformation in Ti64, which is accelerated by Cu diffusion, thereby shifting the transformation kinetics relative to those of the base alloy [5,6]. The pronounced acceleration of densification above 950 °C in high-Cu samples is consistent with literature observations of enhanced liquid-phase sintering efficiency when transient liquids have low viscosity and high surface-tension gradients [3,4,7].

**Figure 2:**
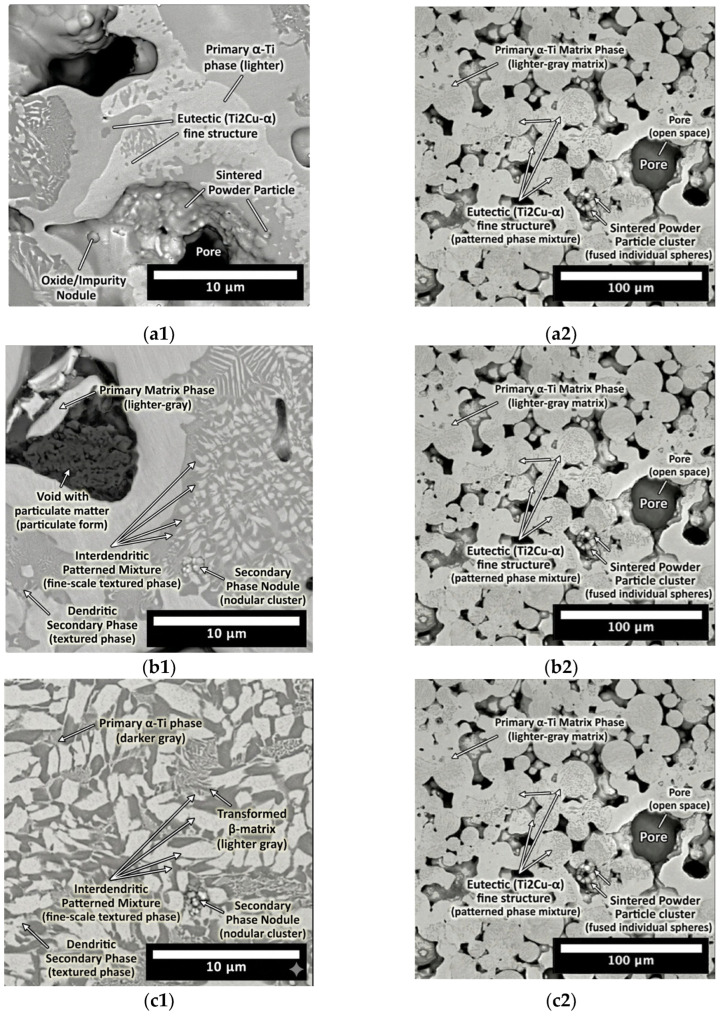

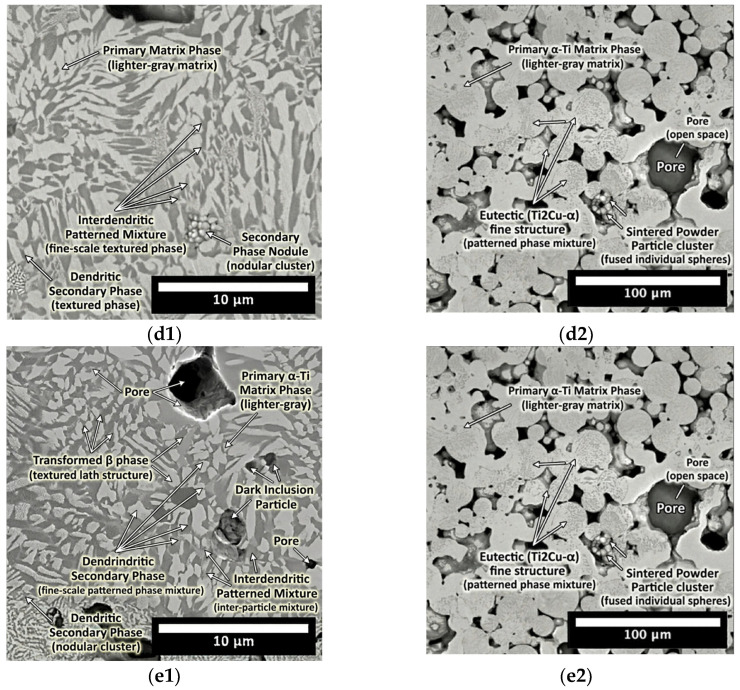
SEM micrographs of Ti6Al4V/20Cu composites sintered at: (**a1**,**a2**) 900 °C, (**b1**,**b2**) 950 °C, (**c1**,**c2**) 1000 °C, (**d1**,**d2**) 1050 °C, and (**e1**,**e2**) 1100 °C.

The green and sintered densities, as well as the average microhardness (Vickers) values of the samples, are listed in Table 1. It was found that the green density (d0) of Ti6Al4V and its composites is very similar, attributed to both powders having analogous morphologies and particle-size distributions at the same weight percentage. However, higher green densities were expected for the composites, given that copper is softer than Ti6Al4V. After sintering, the relative density (ds) increases with the sintering temperature. The sample sintered at 900 °C shows the lowest relative density and microhardness; at this temperature, the transformation begins, but the liquid’s viscosity is insufficient to activate liquid-phase capillarity, so densification is mainly governed by solid-state sintering of Ti6Al4V, with only a minor contribution from liquid diffusion in solid particles. This is consistent with the microstructure observed by SEM in Figure 2a, where interparticle pores and slight neck formation during sintering are evident. Temperatures of 1000 °C and above increase the relative density of the composites to 83.4%, thereby reducing porosity by rounding pores between particles and promoting neck growth, as shown in Figure 2b,c. As previously discussed, samples sintered at 1100 °C achieve densification above 90%, and the pores appear isolated and predominantly spherical (Figure 2d,e). The maximum relative density is around 97% and features small, spherical pores (Figure 2e); however, the liquid distribution around Ti6Al4V particles is not distinguishable in SEM images, in contrast to reports in other liquid-phase sintered systems that rely on eutectic reactions [1] or elemental melting [8]. Densification, calculated as (D_s_ − D_0_)/D_0_ after sintering, is four times higher for the sample sintered at 1100 °C with 20 wt% Cu than for samples treated at 900 and 950 °C. Similarly, microhardness increases with sintering temperature, reaching its maximum at 1100 °C. This suggests that 1100 °C is the optimal temperature for achieving fully densified samples under these sintering conditions.

The densification behavior observed in this study suggests a transition in the dominant sintering mechanism as the temperature increased. At 900–950 °C, densification was primarily controlled by solid-state diffusion between Ti6Al4V and Cu particles, resulting in limited shrinkage and relatively high residual porosity. As the temperature increased to 1000–1050 °C, enhanced Cu diffusion promoted neck growth and interfacial reactions, leading to the formation of TiCu and Ti_2_Cu intermetallic phases. At 1100 °C, where copper is above its melting temperature, densification was further enhanced by liquid-phase sintering mechanisms. Under these conditions, capillary forces generated by the liquid phase promoted particle rearrangement, pore filling, and accelerated mass transport, resulting in the highest relative density and the most homogeneous microstructure. Therefore, the overall densification process can be interpreted as a progression from diffusion-controlled solid-state sintering to capillary-assisted liquid-phase sintering with increasing temperature.

Although SEM observations clearly indicate a progressive reduction in pore size and pore connectivity with increasing sintering temperature, quantitative image analysis of porosity (e.g., pore area fraction, average pore size, and pore size distribution) was beyond the scope of the present study. Therefore, the discussion of pore evolution is based on qualitative microstructural observations. Future investigations incorporating quantitative image analysis will provide a more rigorous correlation between pore morphology, densification, mechanical properties, and corrosion behavior.

### 3.2. Microstructural Analysis

The microstructure of Ti6Al4V/20Cu composites was analyzed using SEM. All samples exhibited a fragmented dendritic structure, producing Ti-Cu globules, which increased with increasing sintering temperature. The sample sintered at 900 °C displayed a lamellar α-β Ti phase, with thin β-Ti lamellae visible as bright features in Figure 2a. The α-Ti phase predominates throughout the remainder of the sample.

Copper addition led to the formation of secondary phases within Ti6Al4V particles, primarily composed of Ti and Cu, which contributed to densification but exhibited limited diffusion within the Ti64 matrix. Copper mainly promoted particle rearrangement, surrounding particles, and preferentially occupying interparticle pores and neck regions formed during initial Ti64 solid-state sintering via capillarity-driven dendrite arm fracture. As the sintering temperature increased to 950 °C, β-Ti lamellae were no longer detected, and the microstructure consisted of Ti-Cu metallic compounds dispersed in an α-Ti matrix (see Figure 2b). At this point, copper began to fill pores, with Ti-Cu layers surrounding particles that had not fully diffused into the matrix, and the amount of dendritic globules increased. At 1000 °C, copper showed greater diffusion within the Ti64 matrix, and Ti-Cu phases distributed more homogeneously in the α-Ti matrix, with the Ti-Cu layer no longer visible, giving way to a fully eutectic structure of fragmented, globular Ti-Cu dendrites formed through semi-solid fusion and Ti matrix arm diffusion, where increased liquid activity enhanced densification. Although copper diffusion increased significantly at temperatures above 1000 °C, complete dissolution of Cu into the Ti matrix was not achieved. SEM/EDS observations together with XRD analysis confirmed the persistence of Cu-rich intermetallic phases, primarily TiCu and Ti_2_Cu. Therefore, the microstructural evolution is better described as a combination of copper diffusion into the Ti matrix and the concurrent formation of intermetallic phases, rather than as complete dissolution of copper.

As the temperature rose, the microstructure evolved and refined, especially at 1100 °C, where residual solid-state sintering pores collapsed due to Cu diffusion into Ti. The microstructure comprised fragmented dendrites that formed elongated Ti-Cu globules, embedding progressively finer features in the α-Ti matrix up to 1050 °C (see Figure 2d1). Above 1100 °C, the microstructure stabilized, showing a more homogeneous eutectic structure and the presence of globules, consistent with a proposed recrystallization mechanism by Vogel [9]. Elevated liquid activity from temperature differences between elements promoted plastic deformation and dislocation generation during grain formation, with molecular migration and coalescence forming grain boundaries. When orientation differences between grains exceeded 20º, the grain-boundary energy surpassed that of the solid–liquid interface, enabling rapid liquid penetration and branch separation to form globules, as described in Kirkwood’s model [10].

**Figure 3:**
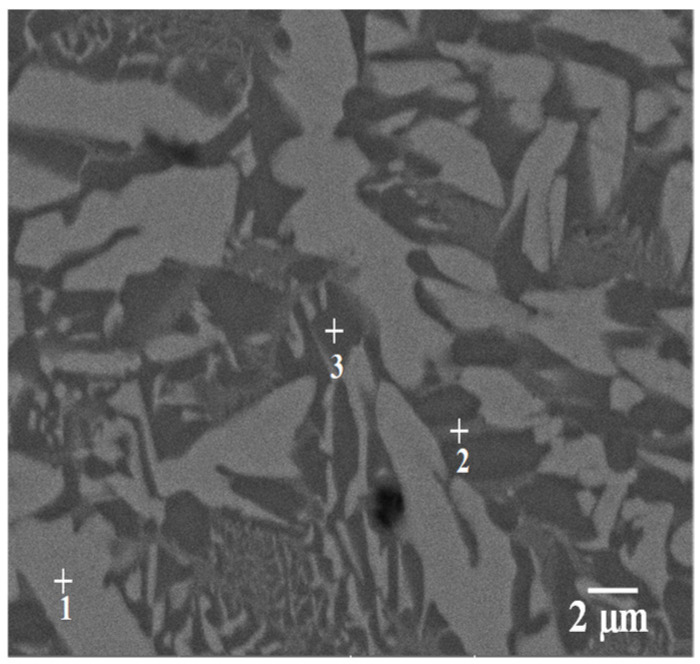
Ti64–20Cu sample sintered at 1100 °C.

To determine the phase composition, EDS spot analyses were performed at the three locations indicated in Figure 3. As observed, Spot 1 is located within the Ti–Cu lamellar region. The composition measured at Spot 1 (63.25 at.% Ti and 34.33 at.% Cu) is closer to the stoichiometric composition of Ti_2_Cu (66.7 at.% Ti and 33.3 at.% Cu) than to TiCu (50 at.% Ti and 50 at.% Cu), see Table 2. Therefore, this region is more appropriately interpreted as a Ti_2_Cu-rich intermetallic area rather than a single TiCu phase. Nevertheless, considering the coexistence of TiCu and Ti_2_Cu phases identified by XRD, the analyzed region may also contain contributions from both intermetallic compounds. This interpretation is consistent with the phase evolution observed during sintering and with the Ti–Cu phase diagram [1]. The composition identified at Spot 2 shows a lower Cu content, which may indicate the formation of coarser lamellae driven by Cu diffusion from smaller lamellae formed during the early stages of the eutectic reaction, see Table 2. Finally, the composition measured at Spot 3 exhibits an even lower Cu content, suggesting that this region consists predominantly of the Ti6Al4V matrix with a slightly increased Al concentration, see Table 2. This observation is consistent with the α-Ti phase acting as the matrix, since Al is a well-known α-phase stabilizer.

**Figure 4:**
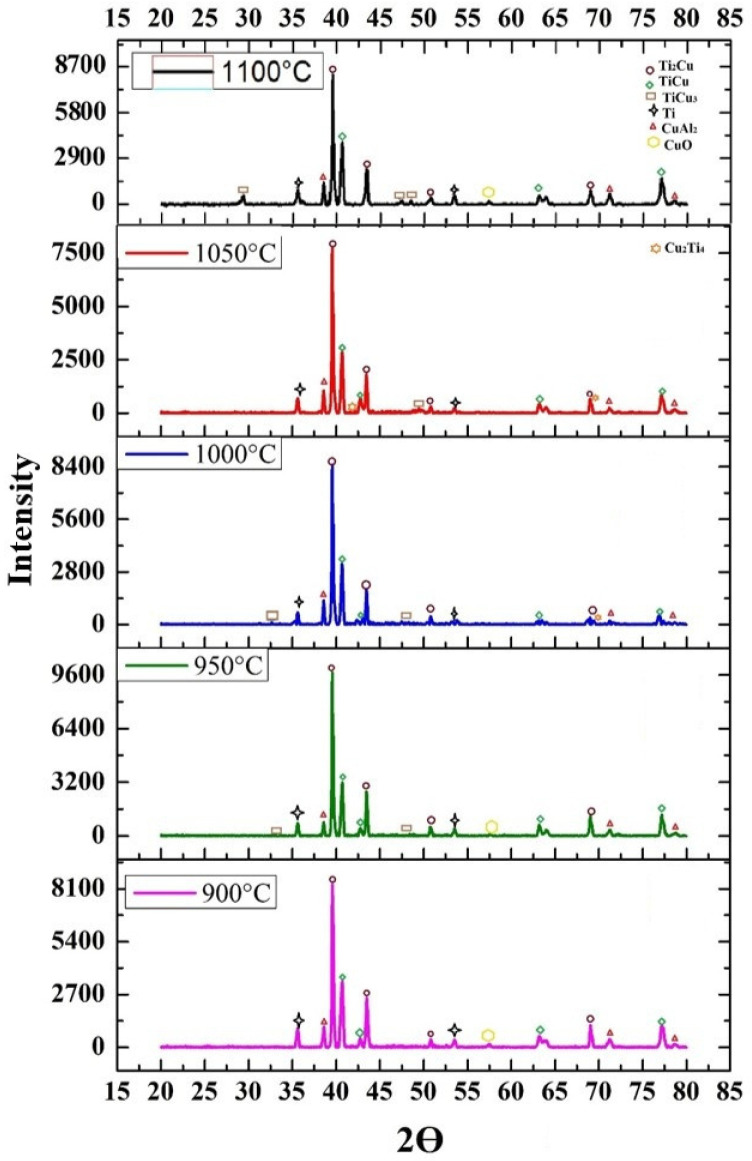
X-ray diffraction patterns of Ti6Al4V/20Cu samples sintered at different temperatures.

The X-ray diffraction patterns of the samples sintered at 900–1100 °C are shown in Figure 4. The characteristic phases were identified, and with increasing sintering temperature, the main peaks of Cu and the intermetallic compounds CuTi and Ti_2_Cu, previously reported elsewhere, become more evident [11]. The main TiCu peak at 39° confirms that most of the Cu remained alloyed with Ti6Al4V after sintering. The simultaneous detection of TiCu and Ti_2_Cu diffraction peaks indicates that part of the copper reacted with titanium to form stable intermetallic compounds during sintering. Consequently, copper was partitioned between the Ti matrix and intermetallic phases, rather than being completely dissolved into the α-Ti matrix. These results indicate that Cu diffusion into Ti is promoted during liquid-phase sintering by increased temperature and the formation of Al–Cu compounds, driven by capillary forces associated with reduced liquid Cu viscosity. From 950 °C onwards, the metastable TiCu_3_ phase was detected and became more pronounced with increasing sintering temperature. This behavior may be associated with enhanced Cu diffusion and an increased fraction of the liquid phase during sintering. The formation of TiCu and Ti_2_Cu phases is considered beneficial, as previous studies have linked these intermetallic compounds to improved antibacterial performance and corrosion resistance in Ti–Cu systems [12,23,24]. Nevertheless, antibacterial activity was not evaluated in the present work; therefore, no direct conclusions can be drawn regarding biological performance.

### 3.3. Mechanical Test

#### Compressive Mechanical Behavior

**Figure 5:**
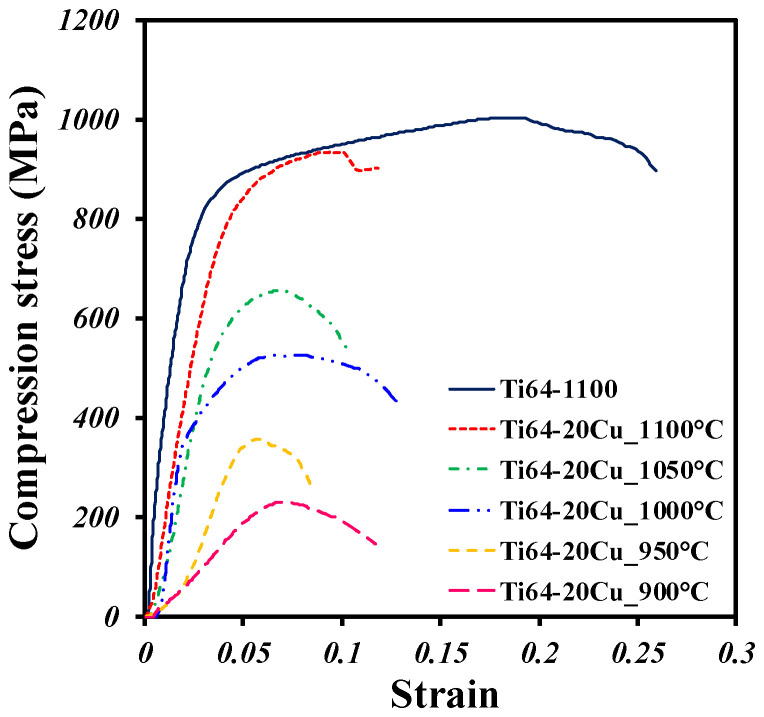
Compressive stress–strain curves for Ti64 and Ti64/20Cu composite samples sintered at different temperatures.

The effect of copper addition on compressive behavior was analyzed through stress–strain curves (Figure 5), which reveal pronounced differences in ductility and strength across sintering temperatures and compositions. Strain-to-failure is markedly reduced in all Cu-containing composites, particularly at 20 wt.% Cu, a trend consistent with previous reports attributing embrittlement to intermetallic phase formation at comparable Cu levels and porosities [7].

The apparent Young’s modulus increased from 4.1 GPa at 900 °C to 24.9 GPa at 1100 °C as densification progressed and residual porosity decreased. These values are lower than those commonly reported for fully dense Ti6Al4V and Ti–Cu alloys (typically above 100 GPa [13] and >110 GPa for SPS-consolidated Ti–Cu systems [14]). The moduli obtained for the 1000–1100 °C conditions fall within or near the range reported for human cortical bone (10–30 GPa) [5,17], whereas the specimens sintered at 900 and 950 °C exhibited substantially lower stiffness. This discrepancy is attributed to the residual porosity inherent in powder metallurgy processing and to the methodology used for strain determination, which was based on crosshead displacement without machine compliance correction or extensometer measurements. Consequently, the reported values should be interpreted primarily as comparative indicators of the evolution of stiffness with sintering temperature rather than as intrinsic elastic constants of the material. Nevertheless, the observed increase in apparent modulus with increasing densification is consistent with the progressive reduction in pore volume and improvement in particle bonding, as well as the 41 GPa value numerically predicted for highly intermetallic Ti–Cu alloys [16]. Although the modulus values obtained in this study should be interpreted with caution, the observed stiffness range remains considerably lower than that reported for fully dense titanium alloys. This behavior is primarily associated with the residual porosity of the sintered materials, which may reduce stiffness and mitigate stress-shielding effects in biomedical applications [17].

The evolution of strength with sintering temperature and Cu content further underscores this compatibility. Yield strength rises from 176 MPa at 900 °C to 682 MPa at 1100 °C, while ultimate compressive strength (UCS) increases from 230 to 934 MPa over the same interval (Table 3). These values exceed those typically reported for traditional porous Ti6Al4V scaffolds (100–400 MPa) [17], although they remain below those of fully dense Ti–Cu alloys produced by pressure-assisted techniques, which can reach ~1800 MPa for Ti/10Cu [25]. Nevertheless, the yield strength at intermediate Cu levels and sintering temperatures closely matches the ~627 MPa reported for sintered Ti/5Cu alloys by Alshammari et al. [7], thereby validating the strengthening trend associated with the formation of Ti_2_Cu and α-Ti–reinforced microstructures combined with reduced residual porosity [18].

The hardness also reflects progressive densification and microstructural refinement as sintering temperature increases. Vickers microhardness increases from 313 HV at 900 °C to 473 HV at 1100 °C, with intermediate values of 331, 349, and 370 HV at 950, 1000, and 1050 °C, respectively (Table 3). This monotonic rise in hardness is consistent with the higher volume fraction and continuity of the Ti_2_Cu intermetallic phase, and with the reduction in pore size and connectivity at elevated temperatures, both of which enhance resistance to localized plastic deformation. The simultaneous increase in hardness and strength, without a proportional rise in stiffness, suggests that microstructural optimization via controlled sintering and Cu addition can decouple strength and modulus, which is particularly advantageous for tailoring implants to match the mechanical response of bone.

Allowable strain, derived from the stress–strain curves, provides a direct metric of the mechanical compatibility of these composites with bone tissue. Although allowable strain decreases slightly at the highest strength levels, the measured values (17.5–20.8 × 10^−3^) remain within the physiological deformation window defined for compact and trabecular vertebrae [5,17]. This combination of bone-like modulus, enhanced strength and hardness (compared to conventional porous Ti6Al4V scaffolds), and physiologically relevant deformation capacity confirms the suitability of Ti6Al4V/Cu composites—notably those sintered at 1050–1100 °C—for orthopedic applications where both durable mechanical integration and reduced stress shielding are critical [17].

### 3.4. Corrosion Tests

**Figure 6:**
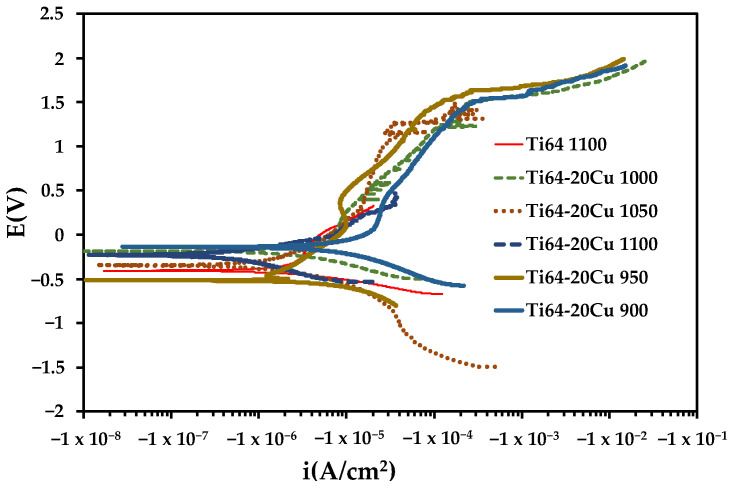
Potentiodynamic polarization curves of Ti64–20Cu samples sintered at different temperatures.

**Figure 7:**
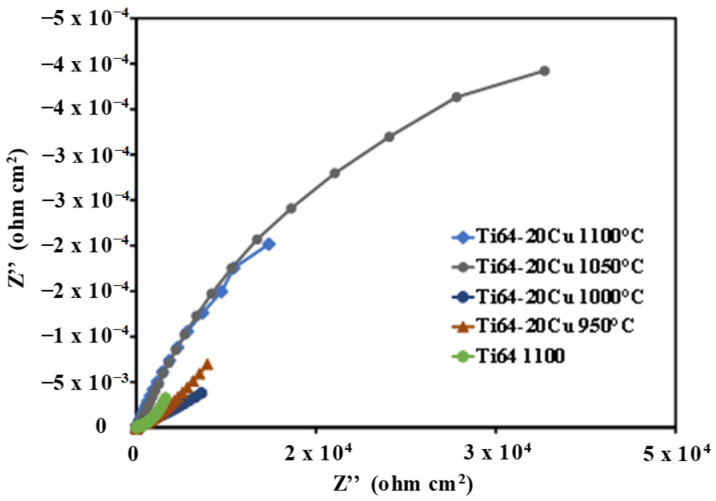
Nyquist plots of Ti64–20Cu samples sintered at different temperatures.

To evaluate the corrosion behavior of Ti64/20Cu samples sintered at different temperatures, potentiodynamic polarization curves (Figure 6) and Nyquist plots (Z′ vs. Z″) (Figure 7) were analyzed. The corrosion parameters are summarized in Table 4. The Ti64 and Ti64/20Cu samples sintered at the same temperature exhibited similar Ecorr values around −0.21 V; however, changing the sintering temperature induced a more electronegative behavior, with an Ecorr of −0.517 V for the Ti64/20Cu sample sintered at 950 °C, which became less negative as the temperature increased, except for the sample sintered at 1000 °C, where a value of −0.185 V was obtained. The lowest corrosion rate (Vcorr) was observed for the sample sintered at 1100 °C (0.0031149 mm/year), and the highest for the sample sintered at 1000 °C (0.019673 mm/year). An interesting observation is that the specimen sintered at 1000 °C exhibited the highest corrosion rate despite presenting a higher relative density than the samples sintered at 900 °C and 950 °C. This behavior suggests that corrosion resistance was not governed exclusively by densification. At 1000 °C, SEM and XRD analyses indicate the presence of an evolving microstructure characterized by increased copper diffusion and the formation of TiCu and Ti_2_Cu intermetallic phases. The heterogeneous distribution of these phases may promote localized micro-galvanic interactions between Cu-rich regions and the α-Ti matrix, accelerating electrochemical activity. As the sintering temperature increased to 1050 °C and 1100 °C, the microstructure became more homogeneous, porosity was further reduced, and intermetallic phases were more uniformly distributed, contributing to the formation of a more stable passive surface layer and improved corrosion resistance. Conversely, the highest Rp value was obtained for the sample sintered at 1100 °C and the lowest for that sintered at 1000 °C, with 9.35 × 10^4^ and 1.94 × 10^4^ Ω·cm^2^, respectively. Another important effect is that the current density in the anodic branch was nearly constant for the Ti64 sample, indicating stable passive behavior due to the formation of an oxide film [19]. This trend aligns with Zhang et al. [7], who reported that adding Cu to porous Ti enhances antibacterial properties without compromising corrosion resistance. This effect was corroborated by analyzing the anodic current density, which showed only a slight increase at higher overpotentials due to the formation of a passive oxide layer. In addition, in Figure 7, the Nyquist plots exhibited a single depressed capacitive semicircle, indicating that charge-transfer reactions at the metal/electrolyte interface predominantly controlled the corrosion process. Such behavior is commonly represented by a simplified Randles-type equivalent circuit consisting of the solution resistance (Rs) connected in series with a parallel combination of charge-transfer resistance (Rct) and a constant phase element (CPE), which accounts for the non-ideal capacitive behavior of the passive surface film. Although no formal equivalent-circuit fitting was performed in the present study, the increase in semicircle diameter with increasing sintering temperature suggests an increase in charge-transfer resistance and consequently improved corrosion resistance [21]. Therefore, adding 20 wt% Cu and sintering at 1100 °C can be considered the optimal processing condition compared with Ti64 sintered at the same temperature and with Ti64–20Cu sintered at lower temperatures.

Copper-ion release is an important aspect of Ti–Cu biomaterials because it influences both antibacterial performance and biocompatibility. Previous studies have reported that Ti–Cu alloys can release Cu ions at concentrations sufficient to inhibit bacterial adhesion and growth while maintaining acceptable cytocompatibility [21,26]. Furthermore, TiCu-containing materials produced by powder metallurgy have demonstrated antibacterial activity attributed to Cu-rich intermetallic phases [23]. In the present study, copper-ion release was not experimentally evaluated; however, the presence of TiCu and Ti_2_Cu intermetallic phases, as identified by XRD and SEM/EDS, suggests that controlled Cu-ion release may occur under physiological conditions. The amount and kinetics of ion release are expected to depend on the volume fraction, distribution, and electrochemical stability of these phases. Nevertheless, this hypothesis requires direct experimental verification. Therefore, no conclusions regarding antibacterial performance or cytocompatibility can be drawn from the present study. Future investigations should include Cu-ion release measurements alongside in vitro biological assessments to establish the relationships among corrosion behavior, antibacterial activity, and biocompatibility.

## 4. Conclusions

This systematic investigation of sintering temperature effects (900–1100 °C) on Ti6Al4V/20Cu composites establishes critical processing–structure–property relationships governing liquid-phase sintering behavior. The results demonstrate that liquid-phase sintering of Ti6Al4V/20Cu composites is fundamentally governed by Cu diffusion kinetics and capillary-driven densification mechanisms, with 1100 °C identified as the optimal sintering temperature for achieving near-full densification (98.1% relative density), representing a fourfold increase in the densification factor compared to lower temperatures. Microstructural characterization reveals progressive evolution from fragmented dendritic structures at 900 °C to refined, globular Ti-Cu intermetallics at 1100 °C, driven by capillary forces and plastic deformation at grain boundaries, with enhanced Cu diffusion into the Ti matrix above 1000 °C and the simultaneous formation of TiCu and Ti_2_Cu intermetallic phases, as confirmed by SEM/EDS observations and XRD analysis. This microstructural refinement directly correlates with substantial mechanical property enhancement, as evidenced by microhardness increasing from 313 HV at 900 °C to 473 HV at 1100 °C (51% improvement), reflecting the elimination of porosity and the formation of hard intermetallic phases. Electrochemical characterization demonstrates superior corrosion resistance at 1100 °C (corrosion rate = 0.0031149 mm/year, polarization resistance = 9.35 × 10^4^ Ω·cm^2^), attributable to dense, stable passive oxide films formed during exposure to the corrosive environment. Electrochemical impedance spectroscopy results indicate enhanced passivation behavior and improved corrosion resistance. The achieved combination of near-complete densification, refined microstructure, enhanced hardness, and improved corrosion performance rank these composites as promising candidates for orthopedic and dental implant applications. Although Ti–Cu intermetallic phases have been associated with antibacterial activity in previous studies, no antibacterial evaluation was performed in the present investigation. These findings provide fundamental insights into processing-controlled densification mechanisms and establish pressureless sintering of Ti6Al4V/20Cu at 1100 °C as a viable route for industrial-scale production of load-bearing biomedical devices with enhanced functional properties.

## Figures and Tables

**Table 1:** The green and sintered densities of all samples, as well as the densification achieved after sintering, were determined.

Temperature (°C)	D_0_ (%)	D_s_ (%)	(D_s_ − D_0_)/D_0_
900	72.8 ± 1.2	78.6 ± 0.5	0.0796
950	72.8 ± 0.9	79.8 ± 0.7	0.0961
1000	72.7 ± 0.7	83.7 ± 1.0	0.151
1050	72.7 ± 1.1	91.4 ± 0.7	0.257
1100	72.7 ± 0.9	98.1 ± 0.3	0.349

**Table 2:** EDS point analysis results for the Ti6Al4V–20Cu sample after sintering at 1100 °C.

Element At. %	Spot 1	Spot 2	Spot 3
Ti	63.25	79.85	85.02
Cu	34.33	5.75	2.81
Al	2.41	10.28	7.99
V	0	4.09	4.17

**Table 3:** Mechanical properties of Ti64/Cu samples sintered at different temperatures.

Sintering Temperature (°C)	E (GPa)	σ_y_ (MPa)	σ_max_ (MPa)	Micro Hardness (HV)
900	4.1 ± 0.8	176 ± 33.4	230 ± 43.8	313 ± 33
950	9.9 ± 1.5	255 ± 40.8	357 ± 57.1	331 ± 39
1000	22.9 ± 3.2	331 ± 46.34	526 ± 76.6	349 ± 27
1050	19.2 ± 2.3	499 ± 59.9	656 ± 78.7	370 ± 14
1100	24.9 ± 2.2	682 ± 61.4	934 ± 84.1	473 ± 20

**Table 4:** Corrosion parameters of Ti64 and Ti64/20Cu samples sintered at different temperatures.

Sample	Ecorr	Icorr	VCorr (mmPY)	Rp
	(V)	(A/cm^2^)		(Ω cm^2^)
TI64–1100	−2.11 × 10^−1^	1.14 × 10^−7^	0.0018369	1.58 × 10^5^
Ti64–20Cu 950	−5.17 × 10^−1^	4.20 × 10^−7^	0.0077675	4.28 × 10^4^
Ti64–20Cu 1000	−1.85 × 10^−1^	9.28 × 10^−7^	0.017162	1.94 × 10^4^
Ti64–20Cu 1050	−3.46 × 10^−1^	4.28 × 10^−7^	0.0079146	4.20 × 10^4^
TI64–20Cu 1100	−2.28 × 10^−1^	1.93 × 10^−7^	0.0031149	9.35 × 10^4^

## Data Availability

The datasets presented in this article are not readily available because the data also forms part of an ongoing study.
